# View-invariant object representation in anterior and posterior inferotemporal cortex: A machine learning approach

**DOI:** 10.1016/j.ibneur.2025.07.010

**Published:** 2025-07-26

**Authors:** Jun-ya Okamura, Daisuke Fukano, Keisuke Murakami, Gang Wang

**Affiliations:** Information Science and Biomedical Engineering Program, Graduate School of Science and Engineering, Kagoshima University, Kagoshima 890-0065, Japan

**Keywords:** Object recognition, View-invariance, Inferotemporal cortex, Machine learning, TE, TEO

## Abstract

Inferotemporal (IT) cortex is the final visual area in the ventral stream where object information is processed. Previous electrophysiological studies showed viewing angle tolerance of 30–60° of single IT cells to the objects experienced in discrimination at each of several viewing angles, and to the objects experienced in learning association of different views. IT is divided into anterior (cytoarchitectonic area TE) and posterior (TEO) parts. It was reported that single cells in area TE showed the viewing angle tolerance while those in area TEO did not. In the present study population activities were compared between cell populations in area TE and those in area TEO using machine learning algorithm. An object set consisted of four similar objects created by deforming a prototype object, and four views each separated by 30°. A population vector was created by aligning responses of the cells to each object image. A classifier was trained by support vector machine (SVM) to create a hyperplane that separated one object from the other three objects at the same viewing angles, and then tested by response vectors to the object images at different viewing angles. In area TE, dynamics of the performance evaluated by d’ showed viewing angle tolerance of 30–90° to the objects with prior experience in learning association of different views. In area TEO, populations of the cells showed the viewing angle tolerance of 30°. Significant increase of the d’ values in area TE in the late time period for the objects with prior experience in learning association of different views may suggest view-invariance is more represented in late time period than early time period. These results suggest that viewpoint invariance is expressed more strongly in the TE region, and expressed in part in the population of the TEO cells.

## Introduction

Retinal images of an object change according to the visual environment, including illumination, distance, position, and viewing angle changes, but we can discriminate objects regardless of the changes. Among these various kinds of changes, viewing angle change is the most challenging problem since the features of an object change drastically as the viewing angle changes. Object information is processed through the ventral stream after the visual information is processed in V1 (Kravitz et al., 2011, Kravitz et al., 2013, Mishkin et al., 1983). Inferotemporal (IT) cortex locates at the final area in the ventral visual stream (Tanaka, 1996). It has been reported that cells in the IT shows size, position, illumination, clutter, and viewing angle tolerance to objects (DiCarlo et al., 2012, Ito et al., 1995, Li et al., 2009, Logothetis and Sheinberg, 1996, Tanaka, 1996). We previously reported that single cells in the anterior part of the IT (cytoarchitectonic area TE) showed viewing angle tolerance of 30–60° to the objects that the monkeys had experienced in discrimination at each of several viewing angles (Okamura et al., 2014). The viewing angle tolerance was comparable to that of the cells responding to the objects experienced by learning association of different views. Populations of the cells in IT showed viewing angle tolerance of up to 90° for the objects experienced by learning association of different views (Yamaguchi et al., 2016). Area TEO is the major afferent to area TE although some afferent from area V4 directly project to area TE (Desimone et al., 1980, Kravitz et al., 2013, Saleem et al., 1993, Ungerleider et al., 2008, Webster et al., 1991). It has been reported in our previous study that single cells in the area TEO does not show the viewing angle tolerance (Okamura et al., 2018). In the present study population responses of the cells in areas TE and TEO were compared by using machine learning algorithm.

An object can be discriminated from the other objects when the viewing angle changes if there are prominent features uniquely representing the object (Biederman, 1987, Hummel, 2001). However, an unfamiliar object cannot be discriminated from similar objects when the viewing angle changes (Bülthoff and Edelman, 1992, Logothetis et al., 1994, Tarr, 1995). Additional learning is required to discriminate similar objects. It was assumed that the view-invariant object recognition develops through experiencing different views of an object and learning association of the different views (Földiák, 1991, Masquelier and Thorpe, 2007, Stryker, 1991, Wiskott and Sejnowski, 2002, Wyss et al., 2006). But, it has been reported that learning association of the different views was not required for the development of the view-invariant object recognition, and that discrimination experience at each of several viewing angles develops the view-invariant object recognition in a viewing angle range of up to 60° (Wang et al., 2005, Yamashita et al., 2010). Subsequent electrophysiological studies showed viewing angle tolerance of TE cells (Okamura et al., 2014, Yamaguchi et al., 2016). The viewing angle tolerance to the objects with prior experience of discrimination at each of several viewing angles was comparable to that to the objects with prior experience of learning association of different views. In the present study, population activities of TE cells to the objects with prior experience of learning association of different views were compared with those of TEO cells, and also compared with the population activities of TE and TEO cells to the objects with prior experience of simple exposure of the object images. The present study aimed to reveal the neuronal mechanisms underlying the development of the viewing angle tolerance through machine learning algorithm, and to reveal the timing of information emergence in neuronal populations of TE and TEO cells.

## Methods

We re-analyzed the data of our previous studies (Okamura et al., 2014, Okamura et al., 2018) that were obtained from monkeys H and M. Data were newly added from monkey M. All procedures using monkeys were performed in accordance with the guidelines of the Japan Neuroscience Society and approved by the Animal Experiment Committee of Kagoshima University.

### Objects

**Fig. 1:**
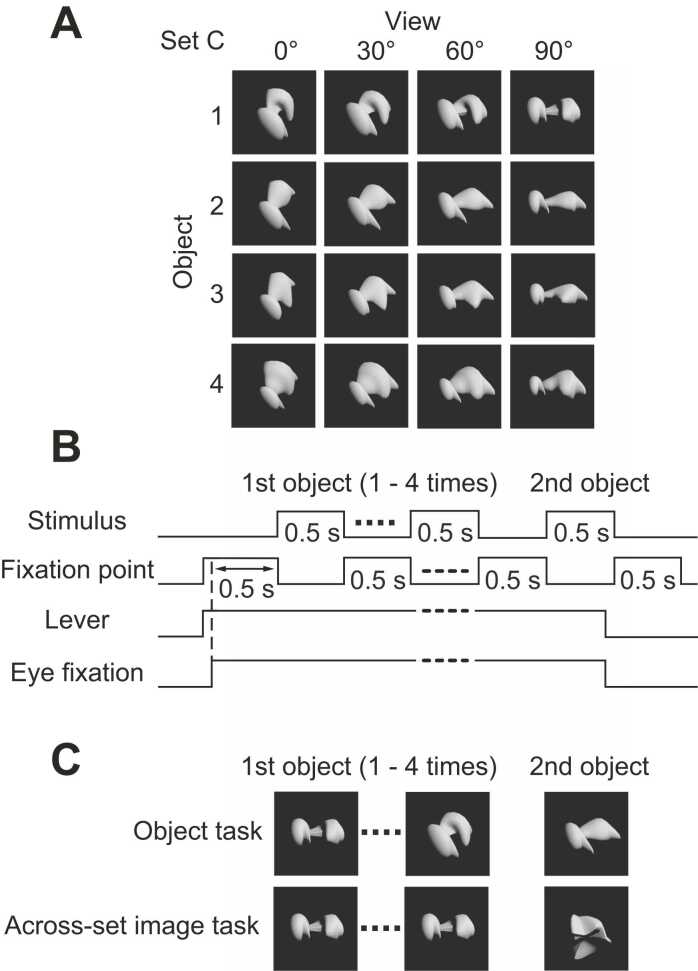
(A) An example of an object set. Four daughter objects were created by deforming a prototype object, and each object was rotated in 30° interval to make a total of 16 object images in an object set. (B) A Basic structure of a task. The task started when the monkey pressed a lever. A first object was presented 1–4 times before the image of a second object was presented. Each image presentation was interleaved with a fixation point. (C) Examples of image presentation. In object task, different views of the first object were presented before the image of the second object selected from the same object set as the first object was presented. In across-set image task, a view of a first object was presented before an image of an object selected from a different object set was presented.

**Fig. 2:**
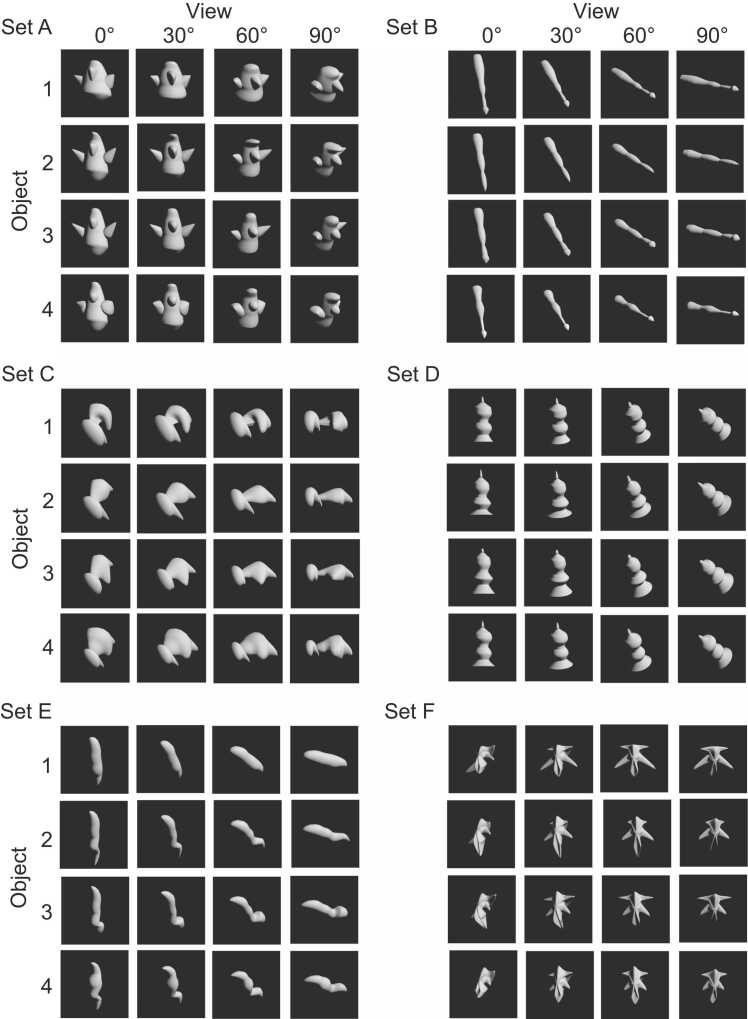
Object sets used in the present study. For explanation see text.

Methods for the object creation were described in our previous papers (Wang et al., 2005, Okamura et al., 2014). In brief, four daughter objects were created by deforming a prototype object in four different directions in three-dimensional space. Six or seven parameters of the object shape such as length, diameter, curvature of a part, and angle between two parts were combined into three parameters that spanned the feature space. Four views were created by rotating the objects in 30° intervals around an axis perpendicular to the axis connecting the viewer’s eyes and the objects (Fig. 1A). A total of six object sets were created by deforming 6 different prototype objects (Fig. 2). The six or seven parameters mentioned above were combined into three parameters, and the amounts were changed to create four daughter objects. The viewing angles of the object images contained in an object set were 0, 30, 60, and 90°. We analyzed the responses of populations of the neurons to the object images at viewing angle differences of 30, 60, and 90°.

### Task

Monkeys were trained to be familiar with the object images with two versions of the task. The task started when the monkeys pressed a lever placed in front of the animal. A first object was presented one to four times before the image of the second object was presented. Monkeys had to release the lever within 1 s after the presentation of the second object (Fig. 1B). In object task, different views of the same object was presented before the image of the different objects within an object set was presented as the second object (Fig. 1C *upper*). In across-set image task, the same view of the first object was presented before the image of an object from different object set was presented (Fig. 1C *lower*). An error with beep occurred when the monkey kept the lever pressed over 1 s after the presentation of the second object, or the monkey released the lever during the presentations of the first object. In the object task, monkeys learned association of different views of an object, while in the across-set image task, monkeys simply experienced each object image. Correct release was defined as the proportion of trials with correct bar releases among the trials in which the second object was presented in the second image presentation. False release was defined as the proportion of trials involving false bar releases among the trials in which the first object was presented in the second image presentation. Discrimination performance was evaluated by using the difference between the proportions of the correct and false releases. After the monkeys became familiar with the tasks using object sets other than those used in the present study, monkeys were trained with the object sets used in the present study. For the across-set image task, monkeys spent 1–2 days to achieve a performance of 0.9–1.0. For the object task, monkeys spent 20–30 days to achieve a performance of about 0.8. Object sets used in the present study were shown in Fig. 2. Object sets were swapped between the two monkeys. Monkey H experienced sets A and B, and sets E and F in object, and across-set image tasks, respectively. Monkey M experienced sets C and E, and sets A and D in object, and across-set image tasks, respectively.

### Electrophysiology

After the discrimination performance was saturated at 0.7–0.9 and number of the presentations of the object images was equal among the object sets, responses of the cells in the areas TE and TEO were recorded. All the images experienced in object and across-set image tasks were presented in across-set image task to exclude the possibility that the task may influence the activities of the neurons. Only across-set image task was used in the electrophysiological recording. The responses to the first image presentation were used for analysis. Details for the electrophysiological recording have been described in our previous reports (Okamura et al., 2014, Okamura et al., 2018). In brief, a tugsten electrode (FHC, USA) was advanced using a micromanipulator (Narishige, Japan) to the inferotemporal cortex. The areas TE and TEO were 16–19 mm and 2–5 mm anterior to the ear bar position, respectively. Single unites were isolated off-line with respect to the shape of the spikes by using Spike sort 3D software (Neuralynx, USA). Spike rates were calculated in 100 ms time windows, which shifted in 20 ms steps. Spontaneous spike rates 500 ms preceding the stimulus onset were subtracted. The spike rates were normalized by the following formula:

$$r_{\mathrm{normalization}}=\frac{r-r_{\mathrm{mean}}}{r_{\mathrm{std}}}$$

The normalized response ($r_{\mathrm{normalization}}$) was obtained by subtracting the mean spike rate of the cell ($r_{\mathrm{mean}}$) from the spike rates (r), and then dividing by the standard deviation ($r_{\mathrm{std}}$). Population vector was created by aligning the responses of individual cells to each image. Individual population vector represents population response to each image. The population vectors for the object images that the monkeys had experienced in object task were pooled, and the population vectors for the object images that the monkeys had experienced in across-set image task were pooled for the monkeys H and M, respectively. For the results of combined monkeys, the population vectors for the object images that the monkeys had experienced in object task were pooled, and the population vectors for the object images that the monkeys had experienced in across-set image task were pooled. Support vector machine (SVM) was used to make a hyperplane that divided an image of an object and the other objects at the same viewing angles. Population vectors in the responses to the images at different viewing angles were used as test. In the training of SVM, one of the four objects was labeled as 1 and the other three were labeled as 0. Population vectors for the object images at a viewing angle difference of 30, 60, and 90° were tested. Discrimination performance was evaluated by using d’, which was calculated from z-transforms of the true and false positives. True positive is a proportion of object images that were correctly labeled as 1. False positive is a proportion of the object images that were wrongly labeled as 1. The z-transforms convert proportions of the true and false positives to z scores. A proportion of 0.5 is converted into a z score of 0. Larger and smaller proportions are converted into positive and negative z scores. The d’ values were calculated in each 100 ms window, which shifted by 20 ms. The analysis was repeated five times by reassigning data for training and testing to get twenty-five d’ values. The d’ values at the same viewing angles were 2.53 and 2.28 for the objects with prior experiences of object and across-set image tasks, respectively, for 60 and 49 TE cells. For 40 and 65 TEO cells respectively responding to the objects experienced in object and across set image tasks, the d’ values at the same viewing angles were 2.09 and 2.58.

### Statistics

Spike rates during the stimulus presentation were compared with those during 500 ms preceding the stimulus using Wilcoxon signed-rank test with Bonferroni correction. Data with p < 0.05 were considered statistically significant. The significance of d’ was evaluated by comparing the d’ values with those obtained by randomly labeling the object images (random labeling). The random labeling was repeated five times by reassigning training and testing data to get twenty-five d’ random labeling values. The significance was evaluated by Mann-Whitney *U* test. P values were obtained in each time bin. We analyzed the data from 450 ms before stimulus onset to 950 ms after stimulus onset in an interval of 20 ms. There were 71 bins. The prior experiences were object and across-set image tasks, the cells were recorded from TE and TEO, and the viewing angle differences were 30, 60, and 90°. In total, 852 bins were analyzed. P < 0.001 was considered statistically significant. When the P values below 0.001 continued five consecutive bins, the d’ values in the time course were considered significant.

## Results

In total, 213 and 422 TE cells, and 246 and 340 TEO cells were recorded from monkeys H and M, respectively. Of them, 60 and 49 TE cells, and 50 and 55 TEO cells recorded from monkeys H and M, respectively, showed statistically significant response to at least one image in an object set. For the objects with prior experience in object task, 35 and 25 TE cells, and 18 and 22 TEO cells showed statistically significant responses in the monkeys H and M, respectively. For the objects with prior experience of across-set image task, 25 and 24 TE cells, and 32 and 33 TEO cells showed statistically significant responses in the monkeys H and M, respectively. In total, 109 and 105 cells in areas TE and TEO, respectively, were used for the analysis.

### Viewing angle difference of 30°

**Fig. 3:**
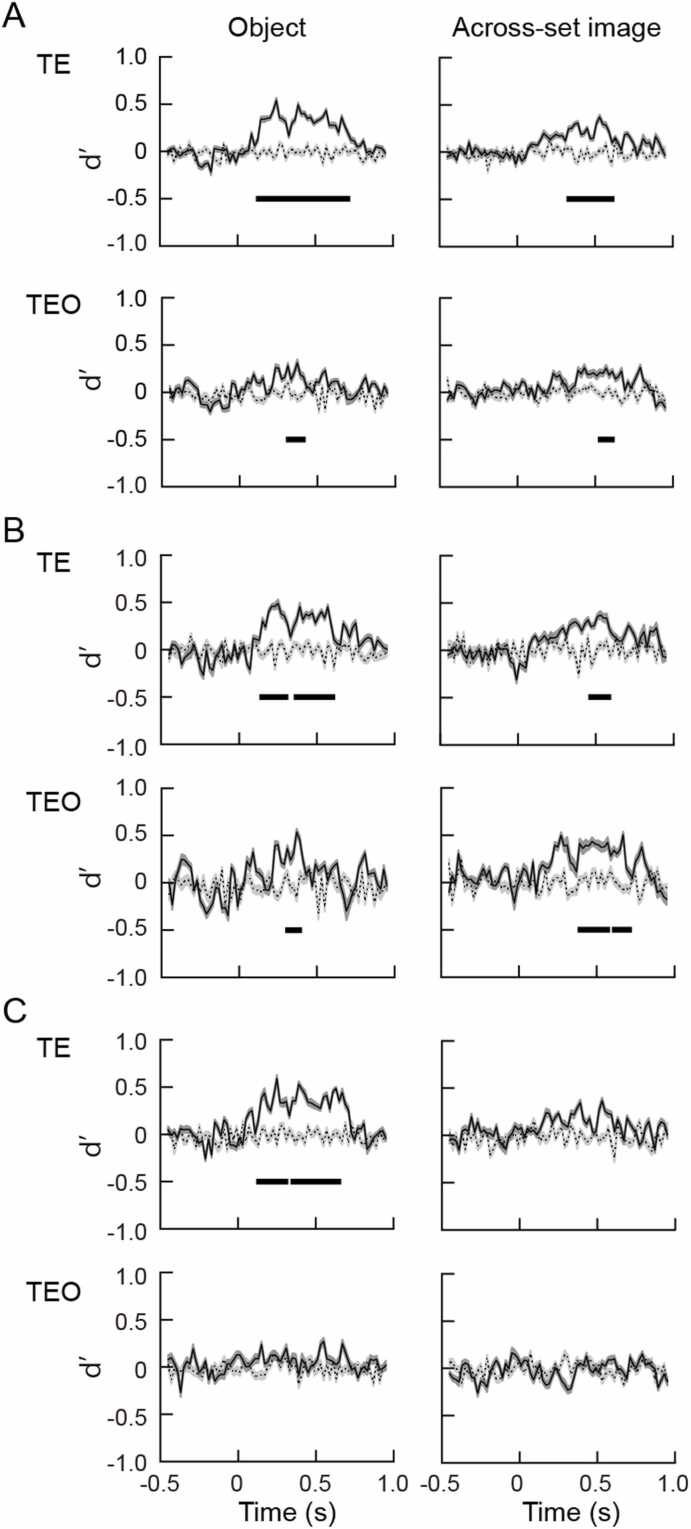
Time courses of d’ values for the objects experienced by the monkeys in object (*left*) and across-set image (*right*) tasks at a viewing angle difference of 30°. Responses recorded from the two monkeys were pooled in A. Responses recorded from individual monkeys were used in B (monkey H) and C (monkey M). In each figures A, B, and C, *upper* and *lower* graphs show d’ values obtained from the population of the cells in areas TE and TEO, respectively. Solid lines and dark gray represent average d’ values and SE of five times repeated analysis, respectively, for the objects experienced in two versions of the tasks. Dotted lines and light gray represent average d’ values and SE of five times repeated analysis, respectively, for the object images that were randomly labeled (random labeling, see text). Thick horizontal bars represent significant differences between the d’ values for the objects experienced by the two versions of the tasks and those for random labeling.

Data of 109 and 105 cells in area TE and TEO were used for analysis. Of them 60 TE cells 40 TEO cells showed statistically significant responses to the object images that the monkeys had experienced in object task. Forty-nine TE and 65 TEO cells showed statistically significant responses to the object images that the monkeys had experienced in across-set image task. A hyperplane was created by training a classifier using SVM. Population response vectors for the object images at a viewing angle difference of 30° were tested. Dynamics of the d’ values of TE and TEO cells are shown in Fig. 3. The d’ values obtained by pooling the responses recorded from two monkeys were significantly different from random labeling at 130–710 ms for the object images experienced in object task, and at 330–610 ms for those experienced in across-set image task (Fig. 3A *upper*). Dynamics of the d’ values of TEO cells are shown in Fig. 3A *lower*. The d’ values were significantly different from random labeling at 310–410 ms in the responses to the object images experienced in object task and at 530–610 ms in the responses to the object images experienced in across-set image task.

Similarly for monkey H, the d’ values were significantly different from random labeling in TE cells at 150–310 ms and 370–610 ms time windows for the objects experienced in object task, and at 470–590 ms for the objects experienced in across-set image task (Fig. 3B *upper*). The d’ values were also significantly different from random labeling in TEO cells at 310–390 ms time window for the objects experienced in object task, and at 390–570 ms and 610–710 ms time windows for the objects experienced in across-set image task (Fig. 3B *lower*). For monkey M (Fig. 3C), significant differences of the d’ values from random labeling were found in TE cells for the objects experienced in object task at 130–310 ms and 350–650 ms time windows, and not in TEO cells (Fig. 3C *left column*). Significant increase of d’ values of TE cells and TEO cells were not found in the objects experienced across-set image task (Fig. 3C *right column*).

### Viewing angle difference of 60°

**Fig. 4:**
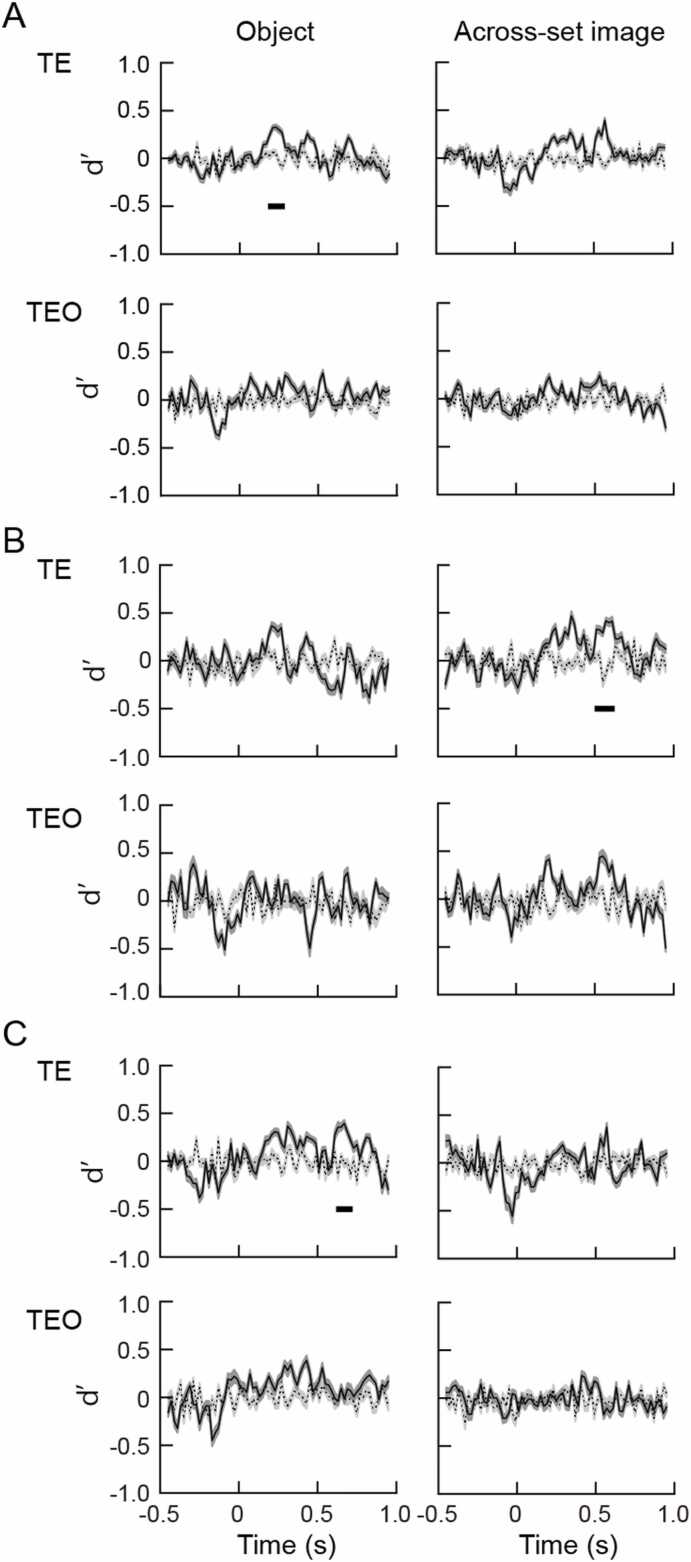
Time courses of d’ values for the objects experienced by the monkeys in object (*left*) and across-set image (*right*) tasks at a viewing angle difference of 60°. Responses recorded from two monkeys were pooled in A, and responses recorded from individual monkeys were used in B (monkey H) and C (monkey M). Notations and layouts are the same as those in Fig. 3.

After a hyperplane was created by training a classifier using SVM, population response vectors for the object images at a viewing angle difference of 60° were tested. Dynamics of the d’ values obtained by pooling the responses of TE and TEO cells recorded from two monkeys are shown in Fig. 4A. In area TE, significant difference between the d’ values for the objects experienced in object task and those for the random labeling was found at 190–270 ms (Fig. 4A *left*). The d’ values were not significantly different from random labeling for the objects experienced in across-set image tasks in area TE (Fig. 4A *right*). In area TEO the d’ values were not significantly different from random labeling for the object images experienced in across-set image and object tasks (Fig. 4A *lower*).

Data were divided for individual monkeys. In monkey H (Fig. 4B), the d’ values were not significantly different from random labeling for the objects experienced in object task, but significantly different at 510–610 time window for the objects experienced in across-set image tasks in TE cells (Fig. 4B *upper*). There were not significant differences between the d’ values and random labeling in TEO cells for the objects experienced in object and across-set image tasks (Fig. 4B *lower*). In monkey M, significant differences of d’ values from random labeling were found at 630–710 ms for the objects experienced in object task (Fig. 4C *left*). The d’ values were not significantly different from random labeling for the objects experienced in across-set image task in TE cells (Fig. 4C *right*). There were not significant differences between the d’ values and random labeling in TEO cells for the objects experienced in object and across-set image tasks (Fig. 4C *lower*).

### Viewing angle difference of 90°

**Fig. 5:**
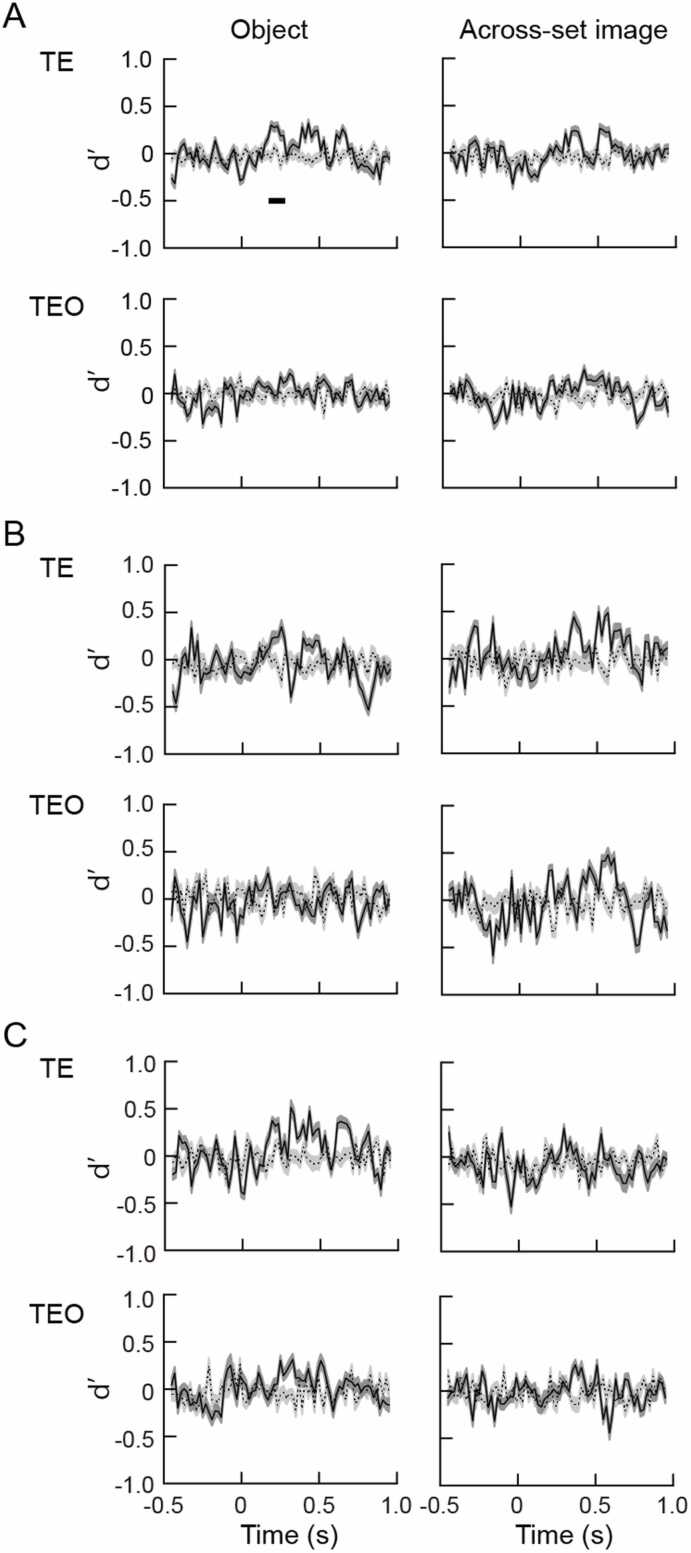
Time courses of d’ values for the objects experienced by the monkeys in object (*left*) and across-set image (*right*) tasks at a viewing angle difference of 90°. Responses recorded from two monkeys were pooled in A, and responses recorded from individual monkeys were used in B (monkey H) and C (monkey M). Notations and layouts are the same as those in Fig. 3.

The d’ values obtained by pooling the responses of TE cells recorded from two monkeys for the object images experienced in object task were significantly different from random labeling at 190–270 time window, but not significantly different for the object images experienced in across-set image task at a viewing angle difference of 90° (Fig. 5A *upper*). The differences between the d’ values and random labeling were not significant in area TEO (Fig. 5A *lower*).

No significant difference between the d’ values and random labeling were found when the data were divided into individual monkeys (Fig. 5B, C).

### Number of cells obtaining reliable d’ values

**Fig. 6:**
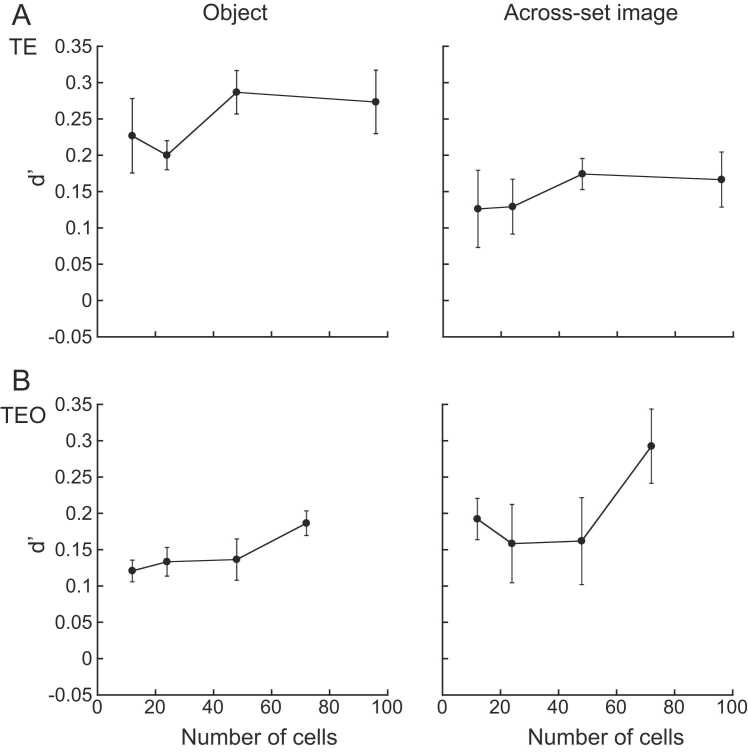
Data at the viewing angle difference of 30° were analyzed by changing the numbers of TE cells (A) and TEO cells (B) included in the analysis. TE cells were randomly selected five times to obtain the numbers of 12, 24, 48, and 96 cells. TEO cells were randomly selected five times to obtain the numbers of 12, 24, 48, and 72 cells. Mean ± se of d’ values were plotted against the numbers of the cells.

Numbers of cells were changed by choosing different numbers of cells in area TE, and TEO (Fig. 6A, and B). The numbers were 12, 24, 48, and 96 for TE cells, and were 12, 24, 48, and 72 for TEO cells. Cells were selected randomly five times. In this analysis spike rates at 100–600 ms time period were used, and population responses to the object images at a viewing angle difference of 30° were tested. The d’ values saturated after the number of cells reached 48 for TE cells. For the objects with prior experience in object task, the d’ values saturated at about 0.28 (Fig. 6A *left*). For the objects with prior experience in across-set image task, the d′ values saturated at about 0.17 (Fig. 6A *right*). Enough numbers of cells were confirmed to be included in the analysis for TE cells. For TEO cells, the d’ values increased as the numbers of the cells increased for the objects experienced in object task (Fig. 6B *left*). For the objects experienced in across-set image task, the d’ values dropped when the numbers of the cells reached 24 and 48, but increased when the number of the cells reached 72 (Fig. 6B *right*). Barely enough numbers of cells were used in the analysis for TEO cells.

**Fig. 7:**
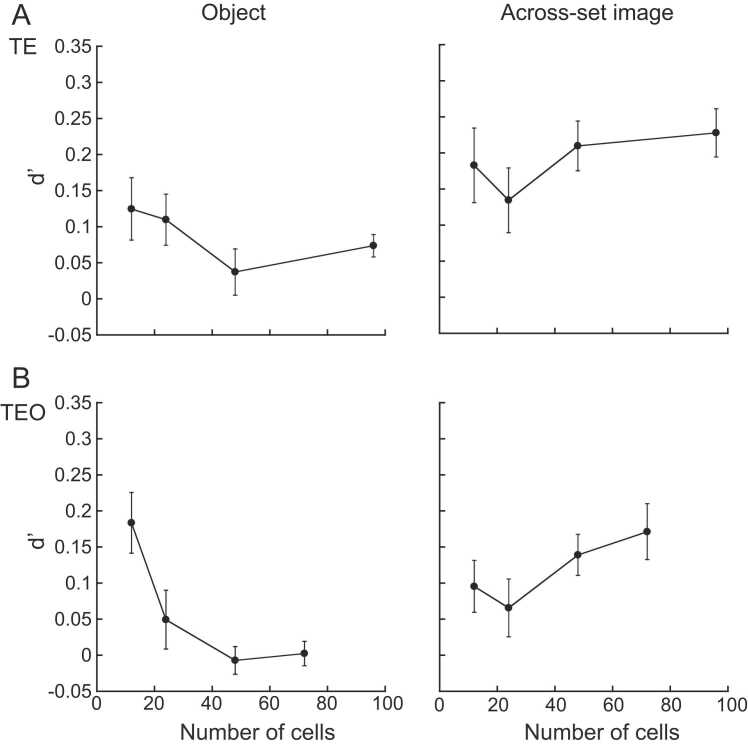
Data at the viewing angle difference of 60° were analyzed by changing the numbers of TE cells (A) and TEO cells (B) included in the analysis. The numbers of the cells, plots, and error bars were the same as those in Fig. 6.

The same analysis was conducted for the object images at a viewing angle difference of 60° (Fig. 7). The results were rather cranky. The d’ values saturated for the objects experienced in across-set image task after the number of the cells reached 48 for TE cells (Fig. 7A *right*). The d’ values dropped for the objects experienced in object task when the number of the cells reached 48 for TE cells (Fig. 7A *left*). The d’ values decreased as the number of the cells increased for the objects experienced in object task for TEO cells (Fig. 7B *left*). The d’ values barely saturated for the objects experienced in across-set image task for TEO cells (Fig. 7B *right*).

## Discussion

In the present study, population responses of the TE and TEO cells were compared using machine learning algorithm. Populations of the TE cells showed viewing angle tolerance in a viewing angle range of 30–90° to the objects experienced in object task, in which learning association of different views of an object was required, but not to the objects experienced in across-set image task, in which monkeys simply experienced object images. On the other hand, populations of the TEO cells showed the viewing angle tolerance to the objects experienced in object and across-set image tasks in a viewing angle range of 30°. In summary, population of TE cells showed significant d’ values in 130–710 ms time period at a viewing angle difference of 30°, showed significant d’ values in 190–270 ms time period at a viewing angle difference of 60°, and showed significant d’ values in 190–270 ms time period for the objects with prior experience of object task. The population of the TE cells also showed significant d’ values in 330–610 ms time period at a viewing angle difference of 30° for the objects with prior experience of across-set image task. Population of TEO cells showed significant d’ values in 310–410 ms time period at a viewing angle difference of 30° for the objects with prior experience in object task. The population of the TEO cells also showed significant d’ values in 530–610 ms time period at a viewing angle difference of 30° for the objects with prior experience in across-set image task. Population of the TE cells showed significant d’ values in a viewing angle range of up to 90° for the objects experienced in object task, and showed significant d’ values in a viewing angle range of 30° for the objects experienced in across-set image task. The d’ values obtained from the data divided for individual monkeys were somewhat different from those obtained from the combined data of two monkeys. The increase of the d’ values indicate objects were discriminable from similar objects, and the object selectivity was robust against changes in viewing angles. The viewing angle tolerance was represented in particular time windows, which is discussed below.

It has been reported that single cells in area TEO do not show viewing angle tolerance to the objects experienced in object and across-set image tasks (Okamura et al., 2018). In the present study, viewing angle tolerance of the population of the cells in area TEO was examined. In the analysis using SVM, population of the cells in area TEO showed the viewing angle tolerance to the objects experienced in object and across-set image tasks in a viewing angle range of 30°. On the other hand, population of the cells in area TE showed viewing angle tolerance at viewing angle differences of 30, 60, and 90° to the objects experienced in the object task. The population of the TE cells also showed viewing angle tolerance at a viewing angle difference of 30° to the objects experienced in across-set image task. It might be possible that viewing angle tolerance would be generated in part by simply viewing each image at the same viewing angles. TEO cells possibly represent the viewing angle tolerance of up to 30° to the objects experienced in object and across-set image tasks as a population, although single TEO cells do not show the viewing angle tolerance (Okamura et al., 2018). In our previous study, neural distance was evaluated by calculating correlation coefficient between the response vectors to the object images experienced in object and across-set image tasks, and subtracting the correlation coefficient from 1 (Yamaguchi et al., 2016). The neural distance between the same object and different objects were significantly different at time windows of 190–890 ms, and 190–850 ms at the viewing angle differences of 30°, and 60°, respectively. At the viewing angle difference of 90°, the neural distance between the same and different objects were also significant at 230–310 ms and 430–550 ms time periods, although the significant time periods delayed and short. In the present study, the criteria for the significance were stricter than those in our previous study (Yamaguchi et al., 2016), and methods for the evaluation of the viewing angle tolerance were different, but the results were consistent with the previous study (Yamaguchi et al., 2016).

In the present study, significant increase of d’ values were obtained in 130–710 ms time period at a viewing angle difference of 30°, in 190–270 ms time period at a viewing angle difference of 60°, and in 190–270 time period at a viewing angle difference of 90° in the population of the TE cells. The beginning of the increase of d’ values were 60 ms longer at a viewing angle difference of 60, and 90° than that at a viewing angle difference of 30° for the objects with prior experience of object task. This might reflect the difference between recognition of objects with viewing angle differences of 30° and 60, 90°. We presented the object images using across-set image task to exclude the possibility that the task may influence the activities of the neurons, and to analyze the response of the neurons to the object images. The reaction time in the across-set image task was 265 ± 29 ms (mean ± SD, n = 50), which was 75–135 ms later than the beginning of the increase of d’ values. To analyze the relationship between the difference of the beginning of the d’ values and reaction time, it may be necessary to present the object images in object task. This point will be answered in more elaborate study.

In the population of the TE cells, the significant increase of d’ values occurred in 150–310 ms and 370–610 ms time periods, and 130–310 ms and 350–650 ms time periods in monkeys H, and M, respectively, at the viewing angle difference of 30°. At the viewing angle difference of 60°, the significant increase of d’ values did not occur in monkey H, but occurred in 630–710 ms time period in monkey M. The significant increase of d’ values occurred in 510–610 ms time period at the viewing angle difference of 60° to the objects experienced in across-set image task in monkey H. The difference between the results obtained with combined data from two monkeys and those obtained with data from single monkeys might be due to the smaller number of neurons included in the analysis of data obtained from single monkeys than that included in the analysis of combined data of two monkeys. Strict results were obtained by using combined data, while incomplete results might be obtained by using data of single monkeys, although significant increases of d’ values were found in the time periods mentioned above.

At the viewing angle difference of 90°, the significant difference was only obtained for the objects that the monkeys had experienced in object task in the present study. The number of components contained in the population vectors differed between the vectors for the object images at viewing angle differences of 30, 60° and 90°. The number of components of the population vectors for the object images at viewing angle differences of 30 and 60° were the same. This difference might cause effects on the d’ values at a viewing angle difference of 90°. More elaborate study is needed to overcome this point.

Responses of the TE and TEO cells were divided into early and late phases (Yamaguchi et al., 2016, Okamura et al., 2018, Dai et al., 2021). At the viewing angle difference of 30°, the d’ values were significantly different from random labeling at 130–710 ms for the objects experienced in object task. For the objects experienced in object task, significant increase of d’ values were found in both early and late phases of the response. The increase of the d’ values in the late phase of the response may support the notion that the late phase might be involved in discrimination of objects regardless of the viewing angle changes (Wang et al., 2021), while the increase of the d’ values in the early phase of the response may reflect the global categorical information, although the viewpoint invariant recognition may start in the middle of the early phase of the response.

It has been reported that responses of TE and TEO cells to the object images contain early and late phases (Sugase et al., 1999, Brincat and Connor, 2006, Tamura and Tanaka, 2001, Matsumoto et al., 2005, Yamaguchi et al., 2016, Okamura et al., 2018). The early phase contains information of global category, or individual parts, while the late phase contains information of fine shape, or facial expression, or specific multipart configurations. It has been reported that initial sensory activities were contained in the early phase of the response, and view-invariant computation may be contained in the late phase of the response (Wang et al., 2021). The results obtained in the present study suggest an idea that view-invariant computation might start in the middle of the early phase of the response.

It was shown that enough numbers of the cells were contained in the analysis of TE cells at the viewing angle difference of 30°. Barely enough numbers were included in the analysis of TEO cells at the viewing angle difference of 30°. The performance increased as the numbers of the cells included in the analysis increased, but did not saturate. We may need to record more numbers of TEO cells for the future study. But, in the current study enough numbers of the cells were included in the analysis for comparing the performance between the populations of the TE and TEO cells. The d’ values fluctuated when the numbers of the cells changed at the viewing angle difference of 60°. The d’ values saturated at the viewing angle difference of 60° for both TE and TEO cells for the objects experienced in across-set image task, but decreased for the objects experienced in object task for both TE and TEO cells. We are not sure whether we obtained enough numbers of the cells for the analysis at the viewing angle difference of 60°. It is probable that we should record responses of more numbers of TE and TEO cells. This point would be answered in more elaborate study.

## CRediT authorship contribution statement

**Jun-ya Okamura:** Writing – original draft, Investigation, Formal analysis, Conceptualization. **Daisuke Fukano:** Software, Investigation, Formal analysis. **Keisuke Murakami:** Software, Investigation. **Gang Wang:** Writing – review & editing, Supervision, Conceptualization.

## Declaration of Competing Interest

The authors declare no conflicts of interest associated with this manuscript.

## References

[bib1] Biederman I. (1987). Recognition by components: a theory of human image understanding. Psychol. Rev..

[bib2] Brincat S.L., Connor C.E. (2006). Dynamic shape synthesis in posterior inferotemporal cortex. Neuron.

[bib3] Bülthoff H.H., Edelman S. (1992). Psychophysical support for a two-dimensional view interpolation theory of object recognition. Proc. Natl. Acad. Sci. U. S. A..

[bib4] Desimone R., Fleming J., Gross C.G. (1980). Prestriate afferents to inferior temporal cortex: An HRP study. Brain Res..

[bib5] Dai L., Okamura J.Y., Wang G. (2021). Dynamics of stimulus selectivity in inferotemporal neurons. Adv. Biomed. Eng..

[bib6] DiCarlo J.J., Zoccolan D., Rust N.C. (2012). How does the brain solve visual object recognition?. Neuron.

[bib7] Földiák P. (1991). Learning invariance from transformation sequences. Neural Comput..

[bib8] Hummel J.E. (2001). Complementary solutions to the binding problem in vision: Implications for shape perception and object recognition. Vis. Cogn..

[bib9] Ito M., Tamura H., Fujita I., Tanaka K. (1995). Size and position invariance of neuronal responses in monkey inferotemporal cortex. J. Neurophysiol..

[bib10] Kravitz D.J., Saleem K.S., Baker C.I., Mishkin M. (2011). A new neural framework for visuospatial processing. Nat. Rev. Neurosci..

[bib11] Kravitz D.J., Saleem K.S., Baker C.I., Ungerleider L.G., Mishkin M. (2013). The ventral visual pathway: an expanded neural framework for the processing of object quality. Trends Cogn. Sci..

[bib12] Li N., Cox D.D., Zoccolan D., DiCarlo J.J. (2009). What response properties do individual neurons need to underlie position and clutter "invariant" object recognition?. J. Neurophysiol..

[bib13] Logothetis N.K., Pauls J., Bülthoff H.H., Poggio T. (1994). View-dependent object recognition by monkeys. Curr. Biol..

[bib14] Logothetis N.K., Sheinberg D.L. (1996). Visual object recognition. Annu. Rev. Neurosci..

[bib15] Masquelier T., Thorpe S.J. (2007). Unsupervised learning of visual features through spike timing dependent plasticity. PLoS Comput. Biol..

[bib16] Matsumoto N., Okada M., Sugase-Miyamoto Y., Yamane S., Kawano K. (2005). Population dynamics of face-responsive neurons in the inferior temporal cortex. Cereb. Cortex.

[bib17] Mishkin M., Ungerleider L.G., Macko K.A. (1983). Object vision and spatial vision: two cortical pathways. Trends Neurosci..

[bib18] Okamura J.Y., Yamaguchi R., Honda K., Wang G., Tanaka K. (2014). Neural substrates of view-invariant object recognition developed without experiencing rotations of the objects. J. Neurosci..

[bib19] Okamura J.Y., Uemura K., Saruwatari S., Wang G. (2018). Difference in the generalization of response tolerance across views between the anterior and posterior part of the inferotemporal cortex. Eur. J. Neurosci..

[bib20] Saleem K.S., Tanaka K., Rockland K.S. (1993). Specific and columnar projection from area TEO to TE ㏌ the macaque inferotemporal cortex. Cereb. Cortex.

[bib21] Stryker M.P. (1991). Temporal associations. Nature.

[bib22] Sugase Y., Yamane S., Ueno S., Kawano K. (1999). Global and fine information coded by single neurons in the temporal visual cortex. Nature.

[bib23] Tamura H., Tanaka K. (2001). Visual response properties of cells in the ventral and dorsal parts of the macaque inferotemporal cortex. Cereb. Cortex.

[bib24] Tanaka K. (1996). Inferotemporal cortex and object vision. Annu. Rev. Neurosci..

[bib25] Tarr M.J. (1995). Rotating objects to recognize them: a case study on the role of viewpoint dependency in the recognition of three-dimensional objects. Psychon. Bull. Rev..

[bib26] Ungerleider L.G., Galkin T.W., Desimone R., Gattass R. (2008). Cortical connections of area V4 in the macaque. Cereb. Cortex.

[bib27] Wang G., Obama S., Yamashita W., Sugihara T., Tanaka K. (2005). Prior experience of rotation is not required for recognizing objects seen from different angles. Nat. Neurosci..

[bib28] Wang R.H., Dai L., Okamura J.Y., Fuchida T., Wang G. (2021). Object discrimination performance and dynamics evaluated by inferotemporal cell population activity. IBRO Neurosci. Rep..

[bib29] Webster M.J., Ungerleider L.G., Bachevalier J. (1991). Connections of inferior temporal areas TE and TEO with medial temporal－lobe structures in infant and adult monkeys. J. Neurosci..

[bib30] Wiskott L., Sejnowski T.J. (2002). Slow feature analysis: Unsupervised learning of invariances. Neural Comput..

[bib31] Wyss R., Konig P., Verschure P. (2006). A model of the ventral visual system based on temporal stability and local memory. PloS Biol..

[bib32] Yamaguchi R., Okamura J.Y., Wang G. (2016). Dynamics of population coding for object views following object discrimination training. Neuroscience.

[bib33] Yamashita W., Wang G., Tanaka K. (2010). View-invariant object recognition ability develops after discrimination, not mere exposure, at several viewing angles. Eur. J. Neurosci..

